# Implementation of deep neural networks to count dopamine neurons in substantia nigra

**DOI:** 10.1111/ejn.14129

**Published:** 2018-09-20

**Authors:** Anna‐Maija Penttinen, Ilmari Parkkinen, Sami Blom, Jaakko Kopra, Jaan‐Olle Andressoo, Kari Pitkänen, Merja H. Voutilainen, Mart Saarma, Mikko Airavaara

**Affiliations:** ^1^ Institute of Biotechnology HiLIFE Unit University of Helsinki Helsinki Finland; ^2^ Biomedicum Fimmic Oy Helsinki Finland; ^3^ Division of Pharmacology and Pharmacotherapy Faculty of Pharmacy University of Helsinki Helsinki Finland

**Keywords:** artificial intelligence, cloud‐based analysis, digital imaging, midbrain, stereology

## Abstract

Unbiased estimates of neuron numbers within substantia nigra are crucial for experimental Parkinson's disease models and gene‐function studies. Unbiased stereological counting techniques with optical fractionation are successfully implemented, but are extremely laborious and time‐consuming. The development of neural networks and deep learning has opened a new way to teach computers to count neurons. Implementation of a programming paradigm enables a computer to learn from the data and development of an automated cell counting method. The advantages of computerized counting are reproducibility, elimination of human error and fast high‐capacity analysis. We implemented whole‐slide digital imaging and deep convolutional neural networks (CNN) to count substantia nigra dopamine neurons. We compared the results of the developed method against independent manual counting by human observers and validated the CNN algorithm against previously published data in rats and mice, where tyrosine hydroxylase (TH)‐immunoreactive neurons were counted using unbiased stereology. The developed CNN algorithm and fully cloud‐embedded Aiforia™ platform provide robust and fast analysis of dopamine neurons in rat and mouse substantia nigra.

## 1 INTRODUCTION

Quantification of cell numbers is a fundamental aspect of biological research. Research in developmental and experimental neurobiology requires unbiased methods to evaluate total amounts of cells in various tissues. Historically neuronal cells have been counted from various samples by visual microscopic inspection with ways to correct quantified cell counts (Abercrombie, 1946; Haug, 1986). With the advent of advanced mathematical models, imaging methods and unbiased computing methods have been successfully implemented in quantifying cells from different brain areas (von Bartheld, Bahney, & Herculano‐Houzel, 2016; West, Slomianka, & Gundersen, 1991). These methods, particularly stereology, are highly time‐consuming (Garcia‐Reitboeck et al., 2013; Ip, Cheong, & Volkmann, 2017; Nair‐Roberts et al., 2008). Moreover, stereology has a certain degree of subjectivity and bias which may be a reason for the variability in results between studies (Ahmad, Park, Radel, & Levant, 2008; Baquet, Williams, Brody, & Smeyne, 2009; Cannon et al., 2009; Elson, Yates, & Pienaar, 2016; German & Manaye, 1993; Marinova‐Mutafchieva et al., 2009; Oorschot, 1996; Runeberg‐Roos et al., 2016; Smeyne et al., 2016).

Stereology with the optical fractionator method and unbiased counting rules has so far been the gold standard to carry out whole brain or brain area neuron counting (Nair‐Roberts et al., 2008; Runeberg‐Roos et al., 2016; Schmitz & Hof, 2005; West et al., 1991). For example, in Parkinson's disease (PD) research, stereology gives good estimates of the total number of dopamine neurons in the substantia nigra pars compacta (SNpc) in rodent PD models (Ip et al., 2017). However, requirement of a stereomicroscope and a suitable counting software limits the use of the method. Thus, there is a need for faster and unbiased analytical methods for neuronal counting.

Machine learning‐based image recognition is an emerging field to classify cells and standardize cell detection to reduce bias and human error. Indeed, supervised machine learning methods enable automated image analysis softwares, such as the widely used CellProfiler (Carpenter et al., 2006). Furthermore, teaching machines to recognize and count cells can reduce the data analysis time significantly. Artificial neural networks (ANN) are a machine learning paradigm inspired by the function of neurons (McCulloch & Pitts, 1943; Patel & Goyal, 2007; Rosenblatt, 1958). More specifically, artificial neural networks are algorithms that function by passing a cascade of subsequent layers of nonlinear processing units to extract structures, features, or patterns from data sets, i.e. are trained to learn from data. Deep neural networks are ANNs that have multiple hidden layers between the standard layers of an ANN, enabling more complex modelling in comparison to similarly adjusted shallow neural networks (Girshick, Donahue, Darrell, & Malik, 2016). Convolutional neural networks (CNN) are ANNs that are especially powerful for pattern recognition in digital images (Fukushima, 1980; Lecun, Bottou, Bengio, & Haffner, 1998). In recent years, the technological advances in graphical processing units have enabled efficient implementation of deep CNNs in biological image analysis with highly promising results (Ciresan, Giusti, Gambardella, & Schmidhuber, 2013; Kraus et al., 2017; LeCun, Bengio, & Hinton, 2015; Mamoshina, Vieira, Putin, & Zhavoronkov, 2016; Turkki, Linder, Kovanen, Pellinen, & Lundin, 2016). Thus, machine learning provides a great possibility to enhance the throughput of analysis of biological samples and to produce new knowledge.

In this study, we aimed to establish CNN‐based quantification of tyrosine hydroxylase positive (TH+) dopamine neurons from rodent substantia nigra. We implemented whole‐slide digital imaging and a cloud‐based image processing and analysis platform (Aiforia™, Fimmic Oy, Helsinki, Finland). On this platform, we trained a first‐in‐class deep CNN algorithm to quantify TH+ neurons in brain sections precisely and efficiently. We first compared the CNN results to manual counts by human observers. Secondly, we selected samples from our previously published studies with results of unbiased stereology from mice (Kumar et al., 2015) and rats (Runeberg‐Roos et al., 2016) for a reanalysis with the neural networks. This is the first report of using a deep CNN to count dopamine neurons in the brain sections. Moreover, a fully cloud‐based platform provides analysis independent of expensive equipment.

## 2 MATERIALS AND METHODS

### 2.1 Animals and surgical procedures

Animals and the surgical procedures have been described earlier (Kumar et al., 2015; Runeberg‐Roos et al., 2016). In brief, 3‐months‐old male glial cell line‐derived neurotrophic factor (GDNF) hypermorphic mice (*n *=* *19) in triple mixed background (129Ola/ICR/C57bl6) (Kumar et al., 2015) and male Wistar rats (220–250 g, *n *=* *44) (Runeberg‐Roos et al., 2016) were housed in 12 hr light/dark cycle at ambient temperature of 20–22°C, 2–5 animals per cage with food and water available ad libitum. The well‐being of the animals was monitored on regular basis. All experimental procedures were conducted according to the EU directive 2010/63/EU on the care and use of experimental animals, and local laws and regulations (Finnish Act on the Protection of Animals Used for Scientific or Educational Purposes [497/2013] and the Government Decree on the Protection or Animals Used for Scientific or Educational Purposes [564/2013]). The experimental design was reviewed and approved by the State Provincial Office of Southern Finland (protocol numbers ESAVI/5459/04.10.03/2011 and ESAVI/3770/04.10.03/2012).

To protect the noradrenergic nerve terminals, desipramine was administered intraperitoneally 30 min before 6‐OHDA (mice 25 mg/kg, rats 15 mg/kg). The 6‐OHDA injection was done under isoflurane and local lidocaine anaesthesia. In mice, 5 μg of 6‐OHDA (Sigma Aldrich, St. Louis, MO) was injected into the right striatum (2 μl, AP +0.7, ML −1.8, and DV −2.7 mm) and buprenorphine was used as a postoperative analgesic (0.1 mg/kg, s.c.). The animals were sacrificed two weeks after the injection. In rats 28 μg of 6‐OHDA was administered to the left striatum (7 μl, AP +1.0, ML +2.8, and DV −6.0, −5.5, −5.0, and −4.4 mm) in four equal depots and 1 mg/kg of tramadol (s.c.) (Orion Pharma, Espoo, Finland) was used as a postoperative analgesic. Two weeks later PBS (10 μl), neurotrophic factors GDNF, or neurturin (NRTN) variants N2 or N4 (each 5 μg) (Runeberg‐Roos et al., 2016) was administered on the same location in a similar manner and the animals were sacrificed 10 weeks later.

### 2.2 Tissue processing and immunohistochemistry

The preparation of tissue sections is described in our previously published studies where the immunohistochemistry is also validated (Mijatovic et al., 2007; Penttinen et al., 2016). In brief, the animals were anesthetized with sodium pentobarbital (100 mg/kg) and perfused intracardially with PBS (mice, (Kumar et al., 2015)) or with PBS and 4% paraformaldehyde (PFA, rats, (Runeberg‐Roos et al., 2016)). The removed brains were postfixed overnight in 4% PFA at 4°C and stored in 20% sucrose. The brains were cut into 40‐μm‐thick sections, the mouse brain in series of three and the rat brain in series of six and stored at −20°C. After quenching the endogenous peroxidase activity with 0.3% hydrogen peroxide (Sigma Aldrich), the free‐floating sections were incubated with 2% normal goat serum and 0.3% Triton X‐100 (mouse) or with 4% bovine serum albumin and 0.1% Triton X‐100 in PBS (rat) to avoid unspecific binding. Next, the sections were incubated overnight with anti‐TH antibody (for mouse sections polyclonal rabbit anti‐TH, MAB152 and for rat sections monoclonal mouse anti‐TH MAB318, Millipore, Billerica, MA, both 1:2000) at +4°C, followed by incubation with the biotinylated secondary antibody (1:200, Vector Laboratories, Burlingame, CA for 1 hour at room temperature). The staining was visualized with 3′,3′‐diaminobenzidine according to the manufacturer's instructions (Vectastain ABC peroxidase kit, Vector Laboratories). Staining and mounting of the sections were done in a blinded manner.

### 2.3 Stereological analysis of TH‐positive cells

The number of TH+ cells was counted using unbiased counting rules with the optical fractionator and dissector principle using StereoInvestigator platform by a blinded observer (MicroBrightfield, Williston, VT) (Kumar et al., 2015; Runeberg‐Roos et al., 2016). In mice, the TH+ cell bodies were analysed from three sections at the medial region of SNpc, around the medial terminal nucleus (approximately from −3.08 to −3.28 mm from bregma) at regular predetermined intervals (*x *=* *100 μm, *y *=* *80 μm, counting frame 60 × 60 μm). In rats, the TH+ cell bodies were analysed from nine sections spanning the whole SNpc (approximately AP −4.5 to −6.0 relative to bregma) at predetermined regular intervals (*x* = 125 μm, *y* = 125 μm, counting frame 80 × 80 μm). The counting frame positions, superimposed on the sample, were randomized by the software. In both cases, the region of interest (ROI) was first defined with 4 ×  objective and the cells were counted with 60 ×  oil immersion objective (Olympus BX51, Olympus, Tokyo, Japan). The coefficient of error was calculated as an estimate of precision and values under 0.1 were accepted.

### 2.4 Cell counting using a deep Convolutional Neural Network

**Figure 1:**
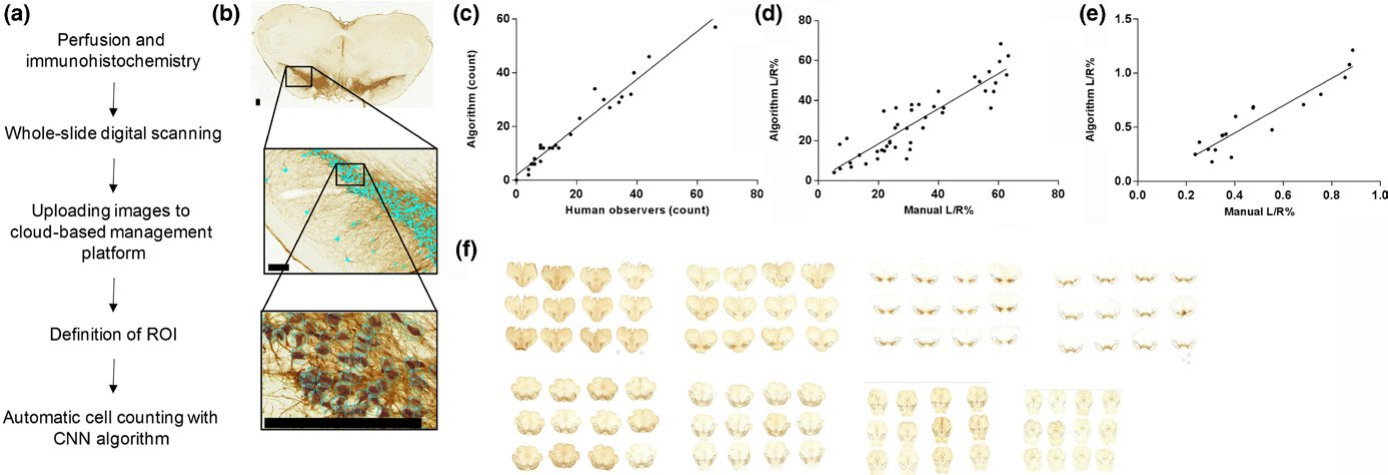
Workflow and validation of the Aiforia™ platform. (a) Schematic diagram of the workflow to implement whole‐slide scanning, cloud‐based image processing, and Aiforia™ platform to count TH‐positive neurons in SNpc. Circles represent detected neuronal somas. (b) Representative figure of the analysed area and CNN performance, scale bar is 100 μm. CNN, convolutional neural network. (c) The algorithm was validated by comparing the results to manual counts obtained by human observers in specific regions in the rat midbrain, *R* ^2^ = 0.95; *y *=* *0.95*x*. (d) The algorithm was next tested against rat samples previously analysed with StereoInvestigator (Runeberg‐Roos et al., 2016). The data is shown as Left (L; lesion side)/Right (R; intact side) ratios; *R* ^2^ = 0.81. (e) The algorithm was tested against mouse samples previously analysed with StereoInvestigator (Kumar et al., 2015). The data is shown as Left (L; lesion side)/Right (R; intact side) ratios; *R* ^2^ = 0.87. (f) 96 consecutive 40‐micron thick brain sections were analysed to obtain the total number of TH‐positive neurons in rat SN. The analysis are is marked in each section. [Colour figure can be viewed at wileyonlinelibrary.com]

For the analysis with a deep neural network, the stained tissue sections were digitized using Pannoramic P250 Flash II whole slide scanner (3DHistech, Budapest, Hungary) with extended focus at a resolution of 0.22 μm/pixel. The extended focus renders the whole section depth in a single focal plane. A total depth of 10 μm was acquired as five focal layers with 2 μm intervals. Next, the digitized images were uploaded to Aiforia™ image processing and management platform (Fimmic Oy, Helsinki, Finland) and analysed using a deep CNN algorithm and supervised learning. The workflow is shown in Figure 1a.

The CNN algorithm was trained to recognize TH+ neuron cell bodies from the digital images in Aiforia^®^ Cloud. The algorithm was trained with 77 megapixels of image data and a total of 1,254 TH+ cell bodies to recognize TH+ neuron cell bodies. The algorithm consisted of two layers. The first layer segmented the TH+ neuron bodies and the second layer counted the individual TH+ cell bodies within the first layer. We used a feature size of 26 μm in the training. The training data for the first layer were digitally augmented for 0°–360° rotation, ±20% size scaling, ±20% aspect ratio change, ±20% shear distortion, ±10% brightness and ±10% contrast change, and the data were flipped both vertically and horizontally. The augmentation parameters for the second layer were 0°–360° rotation, ±20% aspect ratio change, ±10% shear distortion, ±20% brightness and ±20% contrast change, and the data were flipped both vertically and horizontally. The algorithm performance was validated against manual neuron cell body counts by two independent observers in 26 image regions that were not included in the training data.

### 2.5 Comparison of the obtained cell numbers

To compare the total TH+ cell number estimates in rat SNpc obtained with unbiased stereology and CNN, the CNN‐obtained cell numbers of each analysed section was multiplied by six, as the sections were collected at six section intervals. For mouse samples, the CNN cell counts were multiplied by a factor of two as based on their anatomical location of the three counted sections at the anterior posterior line.

### 2.6 Statistical analysis

We used Pearson correlation to analyse the correlation between continuous variables. We calculated precision, recall and F1‐score to compare the neuron counts obtained by human observer and the algorithm (Table 1). The number of true positive, false positive, true negative, and false negative neuron cell body counts were visually counted directly in the digital images.

**Table 1:** Formulas for counting precision, recall and F1‐score for the CNN algorithm

Metrics
Precision = TP/(TP + FP)
Recall = TP/(TP + FN)
F1‐score = 2*Precision*Recall/(Precision + Recall)

FP, false positive; FN, false negative; TP, true positive; TN, true negative.

## 3 RESULTS

### 3.1 CNN algorithm is a reliable method for counting TH‐positive neurons in rat substantia nigra

After the training of the CNN algorithm, we first tested how the CNN algorithm performs against human manual counting. We randomly took regions of interest from rat SN and counted the dopamine neurons manually in the digital images (Figure 1b). Human observers counted a total of 489 TH+ cells and algorithm counted a total of 493 TH+ cells across 26 regions‐of‐interest (ROIs). The correlation between CNN and human counts in terms of counted neurons across the ROIs was very strong (Pearson correlation 0.98, *p *<* *0.001; *R* ^2^ = 0.95; Figure 1c). The performance metrics of the algorithm against human counting are presented in Table 2.

**Table 2:** The results for counting precision, recall and F1‐score of the CNN algorithm versus human observers

	Score (95% Confidence interval)
Precision	88.5% (85.5–91.4%)
Recall	87.8% (84.9–90.7%)
F1‐score	88.2% (85.3–91.0%)

### 3.2 Aiforia™ yields neuron counts comparable to StereoInvestigator in rat and mouse samples

Next, we compared the performance of the Aiforia™ platform against our previous analysis of rat SNpc quantified with StereoInvestigator. Comparison of stereology‐obtained total TH+ cell number estimates and CNN‐obtained estimates (Table 3) demonstrated similar results. In both rat and mouse samples the difference was small, in rats less than 1% difference between estimates with stereology and the algorithm and in mice a 4,8% difference on estimates from the intact hemisphere and a 20% difference in the lesioned hemisphere. Moreover, commonly in the studies using unilateral lesion models the final cell counting results are expressed as a ratio between the lesioned and unlesioned hemispheres. When comparing the ratios obtained with both methods, we found a strong correlation across 44 rat samples (Pearson correlation of 0.9, *p *<* *0.0001; *R* ^2^ was 0.81; Figure 1d). With mouse sections the correlation of the results obtained with these two methods was significant (Pearson correlation 0.93, *p *<* *0.0001 and *R* ^2^ = 0.87; Figure 1e).

**Table 3:** Estimates of total number of TH+ cells in 6‐OHDA lesioned rats and mice with StereoInvestigator (SI) and the CNN algorithm

	CNN lesioned	CNN intact	SI lesioned	SI intact
Rats (vehicle treated)	6,052 ± 1,633	21,443 ± 743	6,000 ± 1,636	21,276 ± 762
Mice (wildtype, vehicle treated)	1,428 ± 180	2,119 ± 95	1,139 ± 147	2,017 ± 100

In CNN analysis the obtained cell numbers were multiplies by six (rat, sections collected at six section intervals, total of nine sections analysed) or two (mouse, sections analyzed at three planes using the medial terminal nucleus of the accessory optic track as anatomical landmark (Kumar et al., 2015). *N *=* *11 in all groups. Data are expressed as mean ± *SEM*.

### 3.3 Analysis of a whole TH‐stained substantia nigra of a rat

Finally, we tested the efficacy of the Aiforia™ platform to perform high‐capacity analysis of rat SN TH+ cells. To quantify the speed of counting we carried out analysis of both hemispheres in 96 consecutive rat brain sections and included both SNpc and SN pars reticulata regions. Thus, the bilateral analysis consisted a total of 192 individual ROIs (Figure 1f). In total, 29 689 TH+ cells were detected and the analysis took 3 hours which equals to detection and counting speed of 2.7 neurons per second. As indicated in the methods, the extended focus scan consisted of 5 layers at 2 μm intervals. This produced the best quality/file‐size ratio, but likely the images missed some cells from the whole 40 μm thick section. Nevertheless, the result is similar to previously reported cell numbers (Oorschot, 1996).

## 4 DISCUSSION

Here, we establish a deep CNN algorithm for counting TH+ cells in SNpc on a fully cloud‐based software platform. The platform allows automatic, fast, and reproducible analysis of cell numbers with minimal hands‐on time. The counting quality of the developed CNN algorithm was good compared to human observers, and the lesion size estimates obtained with automatic CNN analysis correlate well with the estimates obtained with stereology and unbiased counting rules.

Unbiased estimates of cell number in the target structure are an important measure in comparative physiology and neuroscience, among other fields. Stereology with optical fractionator (West et al., 1991) provides high quality estimates of the number of cell bodies. Although the counting process is randomized, the decision of which cells are included in the neuron count is subjective, as the observer bases their decision on the cell shape, size and staining intensity according to the preset rules. However, only small portion of the cells in the target structure are being counted, and the total number of the cells is mathematically estimated. The error of these estimates can be reduced by increasing the number of counting frames leading to laborious and time‐consuming counting procedure. In our recently published study with rats, the average error in the lesion side was 0.098 ± 0.013 and in the intact side 0.049 ± 0.001 (Runeberg‐Roos et al., 2016). With 77 megapixels of training data used for this study, the CNN algorithm error was comparable to the error in stereological counting. However, the performance of the CNN algorithm can be improved with more training.

Several approaches have been developed to count cells. To address the issues of accessibility, usability and the time‐demanding nature of stereological counting, recent developments have improved stereological counting to some extent. It was shown to be possible to use standard light microscopes and free access software such as ImageJ and get similar results to that of a commercial stereology setup (Ip et al., 2017). However, it is approximately 50% more time‐consuming than commercially available stereology platforms. In contrast, one way to reduce time is to use an automated and motorized stage technique, which takes 5–10% less time than stereological method (Tapias, Greenamyre, & Watkins, 2013). Nevertheless, the authors’ model applies mainly the same principles and estimations as the optical fractionator method, opening a possibility for bias. Moreover, the need of physical platform limits access. The common aim of different approaches has been to improve the accuracy of the analysis, reduce the subjective bias and to hasten the analysis. One of the most recent methods is the more developed automatic optical fractionator (Mouton et al., 2017). In this approach, the cells are counted automatically in dissector volumes from extended depth of field images with segmentation algorithm improving the throughput efficiency and reducing the subjectivity bias. Another approach, the proportionator, combines automatic image analysis and nonuniform sampling by dividing sample images in fields of view and counting the cells in these fields (Keller et al., 2013).

Our approach uses a context‐intelligent CNN to count dopamine neurons in SN, which omits the problem of random sampling of counting frames as the algorithm and software platform enable analysis on full SNpc without random sampling. The counting rules were taught to the algorithm which performs the analysis and thus the counting is more uniform and reproducible between sections. The validation showed that the cell numbers obtained with this new algorithm correlates well with the cell numbers obtained by human observer in individual regions of interest. The algorithm counts all the cells within specified ROI at once, reducing the time spent conducting the analysis. In our experience, one brain section (both hemispheres) is analysed within a few minutes. Moreover, the automated counting enables large sample volumes to study even small 10–20% changes. The only possible observer bias after training the algorithm is drawing the outline of the target structure (ROI) in the digital image. According to our experience, the random sampling can be a problem in SNpc. This is mainly because of the shape of the area and uneven TH+ cell density in SNpc. Even with careful definition of the ROI in stereology, the random sampling can lead to a situation where most of the high‐cell density areas are missed reducing the accuracy of the estimation. On the other hand, with stereology, the space between the analysed sections is taken into account, enabling estimation of the total number of cells in the target structures. This feature, however, is not available in the CNN algorithm.

It is important to note that although TH immunohistochemistry is the gold standard to visualize SNpc dopamine neurons, it is well‐known that TH is a readily regulated protein marker. The reduced numbers of TH+ neurons do not necessary mean that the neurons are dead, rather that they have lost their TH+ phenotype (Domanskyi, Saarma, & Airavaara, 2015) or TH has been down‐regulated below the detection level. Because, the reduction in the number of TH‐positive cells does not necessarily indicate cellular death, other plausible strategies such as use of more stable dopaminergic markers vesicular monoamine transporter 2 or dopamine transporter, or a combination of dopaminergic markers with Nissl staining should be considered in assessing the neuronal degeneration.

In conclusion, we present a first‐in‐class, deep CNN algorithm and supervised learning to count dopamine neurons in rat and mouse midbrain anatomical structures. The developed algorithm is fast, reproducible and a precise method to automatically count dopamine neurons in the SNpc requiring minimal hands‐on time. The user‐interface of the CNN algorithm on the Aiforia™ platform is fully cloud‐based and easily accessible to anyone anywhere with an internet connection through any web browser, eliminating the need for microscope equipment to perform the analysis. The novel platform is broadly available for the neuroscience community, and the whole slide scanning can be done with scanners from different manufacturers. In fact, the technology is much more freely available than the current microscope‐based and desktop‐specific software solutions. Moreover, the automated cell counting makes the data collection easier and faster.

## COMPETING INTEREST AND DATA SHARING

The algorithm is proprietary property of Fimmic Oy and the details of the algorithm will not be enclosed. KP is a shareholder of Fimmic Oy and SB is an employee of Fimmic Oy.

## AUTHOR CONTRIBUTIONS

AMP contributed to experimental design and conductions, conceptual design, data analysis and drafting the original manuscript; IP conducted experiments, data analysis and contributed to the drafting of the original manuscript; SB contributed to conceptual design, data analysis, preparation of the figures and drafting the original manuscript; JK also contributed to experimental design, data analysis and editing the manuscript; JOA contributed to experimental design and editing the manuscript; KP, conceptual design, editing the manuscript; MS, conceptual design, funding and editing the manuscript; MHV conceptual design and editing the manuscript; MA, experimental and conceptual design, funding, preparation of the figures and drafting the original manuscript.

## Data Availability

Raw data can be obtained from the corresponding author.
