# Supplementary material for: Implementation of deep neural networks to count dopamine neurons in substantia nigra
Source: Eur J Neurosci. 2018 Sep 20;48(6):2354–61. doi: 10.1111/ejn.14129 (PMC6585833; doi:10.1111/ejn.14129)
Supplement: Supplementary file 1 [file EJN-48-2354-s001.docx]

Implementation of Deep Neural Networks to Count Dopamine Neurons in Substantia Nigra.

Airavaara, Mikko; Penttinen, Anna-Maija; Parkkinen, Ilmari; Blom, Sami; Kopra, Jaakko; Andressoo, Jaan-Olle; Pitkänen, Kari; Voutilainen, Merja; Saarma, Mart

_____________________________________________________________________________________

| Review timeline: | Submission date: 16/03/18  Editorial Decision: Minor Revision  Revision Received: 24/07/18  Accepted: 10/08/18 |  |
| --- | --- | --- |
|  |  |  |

Editor: Panayiota Poirazi

Reviewer 1: Barry Hoffer

_____________________________________________________________________________________

1st Editorial Decision	29/05/18

_____________________________________________________________________________________

Implementation of Deep Neural Networks to Count Dopamine Neurons in Substantia Nigra

Dear Dr. Airavaara,

First please accept our apologies for taking so long to handle your manuscript. The reason was that one of our referees had a serious accident and while he initially promised to return an official review, in the end this was not feasible. However, your manuscript has been reviewed by one external referee (comments below), the section editor P. Poirazi and ourselves. The consensus it that your manuscript is interesting and merits publication at EJN pending some revisions as listed in the report below. We thus look forward to receiving your revised manuscript.

Please also attend to the following issues identified by our editorial office:
2. We would like to publish this work as a technical spotlight
3. To do so, it will be key to fully and comprehensively disclose the algorithm
4. Some of the in-text citations are not in appropriate format and will need to be fixed
5. Last paragraph of results: " In total 29 689 TH+ cells" the 29 seems to be an error  
6. Your data sharing statement needs to be in its own section
7. Your reference list needs to be very carefully checked for missing information
8. Larger, better resolution figure will be needed for publication.

When revising the manuscript, please embolden or underline major changes to the text so they are easily identifiable and DO NOT leave 'track change' formatting marks in your paper.  Please ensure that you provide a text and a figure file for the Graphical Abstract (as detailed in the instructions below).  When carrying out your revisions please refer to the checklist below and visit the EJN author guidelines at www.ejneuroscience.org

When finalized, please upload your complete revised manuscript onto the website, as a Word file (.doc, or .docx).  Please also ensure that a complete set of tables and figures is included as separate files, even if these have not changed from the originals.  At this stage it is necessary to provide high resolution figures.  Please see important instructions below.

Please go into https://mc.manuscriptcentral.com/ejn - Author Centre - manuscripts with decisions where you will find a 'create a revision' link under 'actions'. We ask that you please indicate the way in which you have responded to the points raised by the Editors and Reviewers in a letter. Please upload this response letter as a separate Word (.doc or PDF) file using the file designation "Authors' Response to Reviewers" when uploading your manuscript files.  Please DO NOT submit your revised manuscript as a new one.  Also, please note that only the  Author who submitted the original version of the manuscript should submit a revised version.

If you are able to respond fully to the points raised, we would be pleased to receive a revision of your paper within 30 days.

Thank you for submitting your work to EJN.

Kind regards,

John Foxe &
Paul Bolam
co-Editors in Chief, EJN


Reviews:

Reviewer: 1

Comments to the Author
This is an interesting paper describing a novel computer-based system for counting immunocytochemically defined neurons in brain sections. The counting was compared with more classical unbiased stereology and showed very good correlation with a more rapid analysis with the computer-based system. Clearly, the authors used samples obtained from their previous experiments on rats and mice with 1) unilateral 6OHDA lesions, 2) GDNF family trophic factors, and 3) GDNF hypermorphic mice. However, in this paper, there is no rationale for using these models except for their availability.

It would be interesting, for example, to compare SNpc cell counts on the 6OHDA lesioned vs non lesioned side with this new technology since there is much literature on such comparisons with classical counting methods.

Some other issues:
a) Although TH immunocytochemistry is considered a “gold standard” for SNpc dopamine neurons, it is somewhat problematic as there is both short term (via phosphorylation) and long term (via changes in synthesis) regulation of TH. Moreover, after toxin-induced DA lesions, the SNpc neurons often lose TH phenotype without this cell loss. The authors should comment on this.
b) Were there controls for the TH immunocytochemistry i.e. omission of primary antibody blinded observers etc
c) To what extent would this technology be available to the neuroscience community in terms of cost
d) A small change, on page 5, put “intraperitoneally” after ‘administered’ not 6OHDA

____________________________________________________________________________________

Authors’ Response	06/06/2018

_____________________________________________________________________________________

Dear professors Foxe and Bolam,
Thank you for your response, and decision, and for considering the manuscript for publication. We are sorry to hear about the accident and hope that the referee will recover soon. We have now revised the manuscript according to critic and hope that the manuscript is suitable for publication. The changes to the manuscript have been made with blue font. When the manuscript is accepted, we can try to provide visual abstract.

2. We would like to publish this work as a technical spotlight
Publishing the manuscript as a technical spotlight is fine for us.

3. To do so, it will be key to fully and comprehensively disclose the algorithm
We have revised the methods and have disclosed as many algorithm training parameters as possible. However, the detailed algorithm architecture is developed by company called Fimmic Oy and is their property and trade secret and thus cannot be fully disclosed. We think that this is a common procedure and most of the commercial software code is not open-source (e.g. Microsoft Office products). In fact, even the content of many commercial laboratory reagents/consumables is not disclosed or can be even ill-defined, cell-culture reagent Matrigel being a striking example of these type of products. Manuscript where these have been used are published all the time. Hence, we hope that the data would speak for themselves and the manuscript would be considered for publication without disclosing the algorithm architecture. Moreover, we have revised the text to highlight the fact that the service and algorithms are available for anyone, and from anywhere the globe.

We truly believe and would like to stress that this is a unique time-window to publish data on large-scale, deep-learning based image analytics in neuroscience. This technology and methods will have high impact in all fields of biomedical research in the near future.

4. Some of the in-text citations are not in appropriate format and will need to be fixed
We have fixed these and corrected citations now into EJN format.

5. Last paragraph of results: " In total 29 689 TH+ cells" the 29 seems to be an error
This is not an error, the actual amount counted is two thousand six hundred and eighty-nine cells. This is the amount of an intact, whole adult rat substantia nigra (96 sections, 40 µM each, worth of cells). It should be noted that it is the count of two hemispheres. The purpose of this data was to show the speed of counting. The scanned image did not consist the section throughout, and therefore it is likely that some of the cells were missed. Nevertheless, the count is somewhat close to the numbers reported in the literature.

6. Your data sharing statement needs to be in its own section
We have now changed it, and it is a separate section.

7. Your reference list needs to be very carefully checked for missing information
We have added the missing information.

8. Larger, better resolution figure will be needed for publication.
This is now provided.

Reviews:
Reviewer: 1

Comments to the Author
This is an interesting paper describing a novel computer-based system for counting immunocytochemically defined neurons in brain sections. The counting was compared with more classical unbiased stereology and showed very good correlation with a more rapid analysis with the computer-based system. Clearly, the authors used samples obtained from their previous experiments on rats and mice with 1) unilateral 6OHDA lesions, 2) GDNF family trophic factors, and 3) GDNF hypermorphic mice. However, in this paper, there is no rationale for using these models except for their availability.
We agree with the reviewer. The data was used rather because we analyzed them with StereoInvestigator earlier and we used it to compare the developed method.

It would be interesting, for example, to compare SNpc cell counts on the 6OHDA lesioned vs non lesioned side with this new technology since there is much literature on such comparisons with classical counting methods.
This is an essential part of the validation. As the reviewer pointed out, there is a vast number of studies counting and comparing lesioned vs non-lesioned substantia nigra DA neurons which is why we have compared the 6-OHDA lesioned mice using StereoInvestigator and compared it to the algorithm as can be seen from the results and figure as follows: “To test whether the CNN algorithm yields comparable results against SI in mouse sections, we analysed the neuron counts using CNN in mice treated with 6-OHDA and compared the results against StereoInvestigator. Again, we found that the relative neuron counts (lesion vs intact) strongly correlated between the two methods tested (Pearson correlation 0.93, P<0.0001 and R2 = 0.87).”

We have now also added data (Table 3) about the whole cell counts in the lesioned versus non-lesioned hemispheres and agree with the reviewer that this is an important part to add.

Some other issues:
a) Although TH immunocytochemistry is considered a “gold standard” for SNpc dopamine neurons, it is somewhat problematic as there is both short term (via phosphorylation) and long term (via changes in synthesis) regulation of TH. Moreover, after toxin-induced DA lesions, the SNpc neurons often lose TH phenotype without this cell loss. The authors should comment on this.
Thank you, the problems surrounding TH-staining’s, and we agree with them entirely. They had been overlooked by us, and we have added a discussion regarding this caveat.

b) Were there controls for the TH immunocytochemistry i.e. omission of primary antibody blinded observers etc
Yes, in all the stainings we have validated so that we have omitted primary antibody or secondary antibody. We have also done all the stainings by blinded observers, as well as naturally the algorithm cannot have any bias. We have commented this on the methods.

c) To what extent would this technology be available to the neuroscience community in terms of cost
Thank you, this is an important aspect that we have addressed in the discussion further. In short, the technology is available to everyone as it is a web-based cloud embedded platform. However, the costs are subjective since comparing the price of using the platform to acquiring a commercial stereological platform (SteroInvestigator for example) would deem it relatively inexpensive, but on the other hand, one can do stereology with a standard microscope and free software (ImageJ) (Ip et al., 2017). IN fact, we believe that this technology is much more freely available than for example StereoInvestigator. The whole slide scanners are getting more and more popular, and the counting will be available to everybody at a very reasonable price. Furthermore, it will save time from many talented students to not save hours and hours under a microscope.

d) A small change, on page 5, put “intraperitoneally” after ‘administered’ not 6OHDA
Thank you, it has now been corrected.


On behalf of all authors,
Mikko Airavaara Ph.D.
